# A Multiplex Nanopore Sequencing Approach for the Detection of Multiple Arboviral Species

**DOI:** 10.3390/v16010023

**Published:** 2023-12-22

**Authors:** Joilson Xavier, Vagner Fonseca, Talita Adelino, Felipe C. M. Iani, Glauco C. Pereira, Myrian M. Duarte, Mauricio Lima, Emerson Castro, Carla Oliveira, Hegger Fritsch, Natalia Guimarães, Ludmila O. Lamounier, Fernanda Khouri Barreto, Camilo M. M. Braga de Oliveira, Crhistinne C. Maymone Gonçalves, Danielle Malta Lima, Elaine C. de Oliveira, Gislene G. de Castro Lichs, Iago Gomes, Janaina Mazaro, Janete T. N. Rodrigues, Jayra Abrantes, Jeová K. B. Colares, Kleber G. Luz, Luana Barbosa da Silva, Luiz Demarchi, Magaly C. B. Câmara, Marina C. S. Umaki Zardin, Rafaela Sabatini Mello Pinheiro, Rutilene Barbosa Souza, Simone K. Haddad, Stephanni Figueiredo da Silva, Svetoslav N. Slavov, Themis Rocha, Noelia Morel, Hector Chiparelli, Analía Burgueño, Victoria Bórmida, María N. Cortinas, Rosario S. Martín, Allan C. Pereira, Marcelo F. dos Santos, Walter André Júnior, Jairo Mendez Rico, Leticia Franco, Alexander Rosewell, Rodrigo F. do Carmo Said, Carlos F. C. de Albuquerque, Ethel L. Noia Maciel, Marília Santini de Oliveira, Rivaldo Venâncio da Cunha, Livia C. Vinhal Frutuoso, Ana M. B. de Filippis, Marta Giovanetti, Luiz Carlos Junior Alcantara

**Affiliations:** 1 Instituto de Ciências Biológicas, Universidade Federal de Minas Gerais, Belo Horizonte 31270-901, Brazil; 2 Organização Pan-Americana da Saúde, Organização Mundial da Saúde, Brasília 70800-400, Brazil; 3 Laboratorio Central de Saúde Pública do Estado de Minas Gerais, Fundação Ezequiel Dias, Belo Horizonte 30510-010, Brazilfelipe.iani@funed.mg.gov.br (F.C.M.I.);; 4 Instituto Rene Rachou, Fundação Oswaldo Cruz, Belo Horizonte 30190-002, Brazil; 5 lnstituto Oswaldo Cruz, Fundação Oswaldo Cruz, Rio de Janeiro 21040-900, Brazil; 6 Instituto Multidisciplinar em Saúde, Universidade Federal da Bahia, Vitória da Conquista 45029-094, Brazil; 7 Laboratório Central de Saúde Pública do Acre, Rio Branco 69900-604, Brazil; 8 Secretaria de Saúde do estado do Mato Grosso do Sul, Campo Grande 79031-350, Brazil; 9 Faculty of the Graduate Program in Biotechnology (Renorbio), Universidade de Fortaleza, Fortaleza 60811-905, Brazil; 10 Laboratorio Central de Saúde Pública do Mato Grosso, Cuiabá 78060-628, Brazil; 11 Laboratorio Central de Saúde Pública do Mato Grosso do Sul, Campo Grande 79074-460, Brazil; 12 Laboratório Central de Saúde Pública do Rio Grande do Norte, Natal 59037-170, Brazil; 13 Secretaria Estadual de Saúde do estado do Acre, Rio Branco 69900-064, Brazil; 14 Department of Infectious Diseases, Universidade Federal do Rio Grande do Norte, Natal 59078-900, Brazil; 15 Laboratório de Citogenética, Universidade Federal do Acre, Rio Branco 69920-900, Brazil; 16 Fundação Hemocentro de Ribeirão Preto, Ribeirão Preto 14051-140, Brazil; 17 Departamento de Laboratorios de Salud Pública, Ministerio de Salud Pública, Montevideo 11200, Uruguay; 18 Laboratório de Fronteira Tabatinga, Tabatinga 69640-000, Brazil; 19 Pan American Health Organization, Washington, DC 20037, USA; 20 Secretaria de Vigilância em Saúde e Ambiente, Ministério da Saúde, Brasília 70058-900, Brazil; 21 Coordenação Geral dos Laboratórios de Saúde Pública, Ministério da Saúde, Brasília 70058-900, Brazil; 22 Fundação Oswaldo Cruz, Instituto de Tecnologia em Imunobiológicos, Rio de Janeiro 21040-090, Brazil; 23 Coordenação Geral das Arboviroses, Ministério da Saúde, Brasília 70058-900, Brazil; 24 Sciences and Technologies for Sustainable Development and One Health, Universita Campus Bio-Medico di Roma, 00128 Roma, Italy; 25 Climate Amplified Diseases and Epidemics (CLIMADE), Fundação Oswaldo Cruz, Belo Horizonte 30190-002, Brazil

**Keywords:** nanopore sequencing, arbovirus, Chikungunya virus, orthoflaviviruses, genotyping

## Abstract

The emergence and continued geographic expansion of arboviruses and the growing number of infected people have highlighted the need to develop and improve multiplex methods for rapid and specific detection of pathogens. Sequencing technologies are promising tools that can help in the laboratory diagnosis of conditions that share common symptoms, such as pathologies caused by emerging arboviruses. In this study, we integrated nanopore sequencing and the advantages of reverse transcription polymerase chain reaction (RT-PCR) to develop a multiplex RT-PCR protocol for the detection of Chikungunya virus (CHIKV) and several orthoflaviviruses (such as dengue (*Orthoflavivirus dengue*), Zika (*Orthoflavivirus zikaense*), yellow fever (*Orthoflavivirus flavi*), and West Nile (*Orthoflavivirus nilense*) viruses) in a single reaction, which provides data for sequence-based differentiation of arbovirus lineages.

## 1. Introduction

Infectious diseases caused by viruses transmitted by mosquitoes have been making world headlines since arbovirus outbreaks began appearing in large urban areas. An estimated 3.83 billion people are currently living in areas at risk of dengue, and this number is predicted to increase to 6.1 billion people by 2080, which would represent 60% of the global population in 2080 [1]. Other mosquito-borne diseases, such as Zika and Chikungunya fevers, also represent an important threat to the health and economics of populations around the world, mainly in the region of the Americas, where a total of 3.1 million cases of arboviral diseases were reported in 2022, which represented a relative increase of 118.5% from 2021 to 2022 [2].

Many arboviral diseases lead to clinically indistinguishable febrile syndromes, which makes correct diagnosis challenging. For instance, laboratory differentiation of members of the genus *Orthoflavivirus* (formerly named *Flavivirus*) [3] using serological methods is limited due to extensive cross-reactivity [4]. Nucleic acid tests, such as quantitative reverse transcription polymerase chain reaction (RT-qPCR) tests, have been extensively used for pathogen detection due to their high specificity and sensitivity [5]; however, available tests cannot provide information for differentiating arboviruses at the level of distinct lineages.

DNA sequencing technology has been proven useful in recent efforts to control infectious disease outbreaks, by providing relevant epidemiological aspects regarding the dynamics of an epidemic [6]. The nanopore sequencing platform is a potential tool for diagnostic purposes due to its cost effectiveness, rapid turnaround time, and portability, which allow it to be employed in the investigation of several viral outbreaks [7,8,9]. Hence, in this study, we integrated nanopore sequencing and the advantages of reverse transcription polymerase chain reaction (RT-PCR) to develop a multiplex RT-PCR protocol for the detection of Chikungunya virus (CHIKV) (genus *Alphavirus*) and several orthoflaviviruses in a single reaction, which provides data for sequence-based differentiation of arbovirus lineages.

## 2. Materials and Methods

### 2.1. Primer Design and Selection

We identified a set of primers, previously designed for real-time quantitative reverse transcription PCR (RT-qPCR) [10], which originally amplified a fragment of 260 bases of a conserved region in the NS5 gene of orthoflaviviruses. To take advantage of the long-read sequencing technology of a MinION device, we performed an alignment of 28 reference sequences of common orthoflaviviruses to design a new reverse primer. When used alongside the forward primers (Flavi-all-S and Flavi-all-S2) from a previously published scheme [10], this new reverse primer enabled the amplification of a fragment of around 1000 bases from the NS5 region.

For the detection of the Chikungunya virus’s nucleic acid, we selected a pair of primers previously published for nanopore sequencing [11] and redirected these primers to compose the primer scheme for the optimized protocol described in this study.

We evaluated the specificity of the Chikungunya primers in silico using the National Center for Biotechnology Information (NCBI) Primer-BLAST tool against the NCBI nucleotide collection. However, assessment of the orthoflavivirus primers using the Primer-BLAST tool was not feasible due to its limited ability to accept ambiguity letters beyond “N” within the primer sequences. Consequently, we employed an alternative tool, MFEprimer v.3.0 [11], to conduct in silico checks on both the CHIKV and the orthoflavivirus primers for primer-specific amplification against the NCBI virus database.

### 2.2. Virus and Clinical Specimen Selection

We used virus stocks of Chikungunya virus (CHIKV), as well as *Orthoflavivirus denguei* (DENV) (dengue virus types 1–4), *Orthoflavivirus zikaense* (Zika virus; ZIKV), *Orthoflavivirus flavi* (yellow fever virus; YFV), and *Orthoflavivirus nilense* (West Nile virus; WNV) viruses passaged in C6/36 cells, in L-15 medium kindly provided by the Public Health Laboratory of Minas Gerais state (Fundação Ezequiel Dias) and by the Flavivirus Laboratory of the Oswaldo Cruz Foundation-Rio de Janeiro. Clinical specimens (serum) were provided as residual samples from the epidemiological surveillance routines of the Brazilian Central Public Health Laboratories (LACEN) in Minas Gerais, Rio Grande do Norte, Acre, and Amazonas states. Clinical samples were also obtained from genomic surveillance activities carried out in the Public Health Laboratory of Uruguay and in Ceará state (Universidade de Fortaleza, Brazil). This study was approved by the Pan American Health Organization/World Health Organization (PAHO/WHO) and by the Research Ethics Committee of the Universidade Federal de Minas Gerais, with approval No. 32912820.6.1001.5149. Personal information was de-identified to minimize the risk of the unintended disclosure of the identity of individuals.

Viral RNA, taken from both culture supernatant and clinical specimens, was extracted using the chemagic Viral DNA/RNA 300 Kit H96 (PerkinElmer, Wellesley, MA, USA) according to the manufacturer’s instructions. Extracted RNA was subjected to molecular diagnosis using RT-qPCR to confirm the presence of viral RNA in the samples. Obtained cycle threshold (Ct) values were then used as proxy indicators for the amount of viral genetic material in the screened samples. RT-qPCR tests were performed using the Applied Biosystems 7500 (Thermo Fisher Scientific Inc., Sunnyvale, CA, USA) following previously described protocols [12,13,14,15] for the detection of YFV, ZIKV, CHIKV, and DENV.

### 2.3. Primer Validation using Cultured Viruses and Clinical Specimens

Selected primers were pooled to a concentration of 10 μM and were initially tested using culture isolates of DENV (types 1–4), ZIKV, CHIKV, YFV, and WNV. One-step RT-PCR was performed using the QIAGEN OneStep RT-PCR Kit according to the manufacturer’s instructions, in a total reaction of 25 μL containing 4.7 μL of nuclease-free water, 5 μL of 5x QIAGEN OneStep RT-PCR Buffer, 1 μL of dNTP mix, 6.25 μL of primers (from the pool at 10 μM to a final concentration of 0.5 μM of each primer), 1 μL of QIAGEN OneStep RT-PCR Enzyme Mix, and 7 μL of viral RNA as a template. RT-PCR temperature cycling conditions were as follows: 50 °C for 30 min (reverse transcription); 95 °C for 15 min (initial PCR activation); followed by 40 cycles of 94 °C for 30 s, 55 °C for 30 s, and 72 °C for 90 s; and lastly 72 °C for 10 min (final extension). The same RT-PCR conditions were applied for the validation of the primers using clinical specimens. All reactions contained no-template controls (NTCs) to assess the extent of cross-contamination between neighboring barcoded samples. Electrophoresis in 1% agarose gel was performed to visualize PCR products alongside a GeneRuler 1 kb Plus DNA Ladder (Thermo Fisher Scientific Inc., Sunnyvale, CA, USA). Amplicon concentrations were then quantified using the Qubit fluorimeter with the Qubit dsDNA HS Assay Kit (Invitrogen, Boston, MA, USA).

### 2.4. Nanopore Sequencing

Amplicons were purified using 0.8x AMPure XP beads (Beckman Coulter, Brea, CA, USA), and sample concentrations were normalized to an initial input of 10 ng each. After this point, nanopore library preparation (barcode and adapter ligation) followed the protocol previously described by Quick et al. (2017) [11], which used the Ligation Sequencing Kit (SQKLSK109, Oxford Nanopore Technologies, Oxford, UK) and Native Barcoding Expansion (NBD104 and EXPNBD114, Oxford Nanopore Technologies, Oxford, UK) for the multiplexing of 24 samples. Prepared sequencing libraries were loaded on an R9.4 flow cell, and data were collected for 2 h.

### 2.5. Bioinformatic Analysis for Virus Identification

The raw data (Fast5 files) from the MinION sequencing were basecalled (using the FAST model) and demultiplexed using Guppy v.6.0 (Oxford Nanopore Technologies). Consensus sequence generation, as well as the identification and classification of viral species and lineages [16], were carried out via the Genome Detective software Version 2.41, which uses SPAdes for nanopore single-end reads [17].

The consensus sequences from clinical samples of cases that had tested positive for DENV (types 1–4), CHIKV, ZIKV, and YFV were used to reconstruct the phylogeny of these viruses. Reference sequences were downloaded from NCBI to build separate datasets of partial DENV-2 NS5 (n = 36) and CHIKV envelope (n = 25) genes. After alignment of the sequences generated in this study using MAFFT version 7, these datasets were used to infer the maximum likelihood (ML) phylogenies using IQ-TREE 2.1.1 [18], which also implemented an ultrafast bootstrap (UFBoot) to estimate the statistical support for tree nodes using 10,000 replicates. Distribution charts of reads numbers and RT-qPCR Ct values were built using custom scripts via R studio v.2023.06.1.

## 3. Results

**Figure 1:**
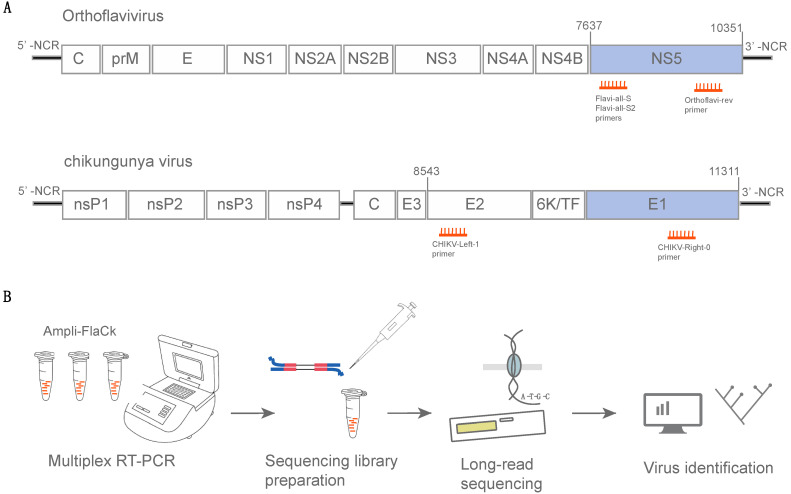
A multiplex RT-PCR protocol for amplification and sequencing of nucleic acids of Chikungunya virus and orthoflaviviruses (Ampli-FlaCk). (**A**) Organization of orthoflaviviruses and Chikungunya virus (genus *Alphavirus*) genomes. Primers (small orange lines) are depicted near their expected binding positions. Genome representations are based on the following references for orthoflaviviruses and Chikungunya virus, respectively: NC_002031 and KP164568.1. (**B**) Schematic representation of the Ampli-FlaCk workflow.

The sequences of the two previously published forward primers [10] were used alongside the new reverse primer (containing degenerate bases in three positions) designed to amplify a fragment of around 1000 bases from a conserved region in the NS5 gene of orthoflaviviruses (Figure 1A). Additionally, a pair of primers was redirected from a published CHIKV sequencing primer scheme [11] to amplify a fragment of ~1100 bases from the CHIKV envelope gene (Figure 1A). We used an in silico approach to assess the specificity of these selected primers by aligning them against the NCBI virus database, which resulted in 8120 potential amplicons covering 20 different virus species when using the orthoflavivirus primers (Table S2). Performing the same analysis using the CHIKV primers resulted in 584 potential amplicons, all from CHIKV isolates. In addition, a Primer-BLAST analysis returned 6084 BLAST hits for Chikungunya virus genomes (see the Data Availability Statement to access the output from the in silico specificity analysis).

These primers (a total of five) were pooled to achieve a multiplex RT-PCR protocol for the amplification of the Chikungunya virus’s and the orthoflaviviruses’ nucleic acids (namely Ampli-FlaCk) in a single reaction. Ampli-FlaCk is able to amplify long amplicons, which, combined with nanopore sequencing, can generate long sequences (~1000 bases) from multiple distinct samples. Then, these sequences can be used for the identification and genotyping of the distinct lineages of Chikungunya virus and orthoflaviviruses (Figure 1B). The nucleotide sequence and physical properties of the primers that comprise the Ampli-FlaCk protocol are listed in Table S1.

**Figure 2:**
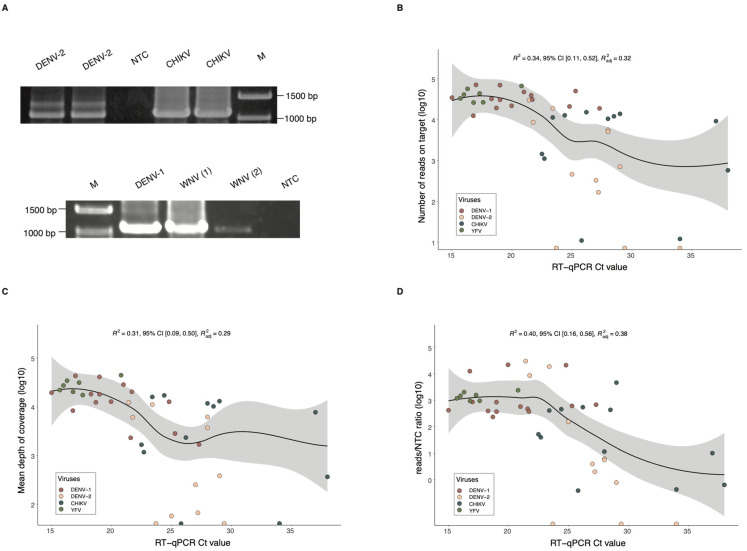
Sequencing performance of the Ampli-FlaCk protocol. (**A**) Electrophoresis analysis of RT-PCR products from cultured dengue virus type 2 (DENV-2, Ct = 22) and Chikungunya virus (CHIKV, Ct = 33), in duplicates (top image). The bottom image displays electrophoresis results for cultured dengue virus type 1 (DENV-1, Ct = 25) and West Nile virus (WNV, numbers in parentheses represent samples having different Ct values, 1 = 21, and 2 = 33). M = Marker. Images were cropped and converted to grayscale for clarity; see the Data Availability Statement for original, unedited images. (**B**) Distribution of the number of reads on target and the RT-qPCR Ct values from the clinical specimens (n = 45) used for testing the protocol. (**C**) Distribution of the mean depth of coverage and the RT-qPCR Ct values from the tested clinical specimens (n = 45). (**D**) Distribution of the reads/NTC ratio and the RT-qPCR Ct values from the tested clinical specimens (n = 45). (**B**–**D**) The dark blue line represents a smooth local regression line (method = LOESS), and the light grey area around the trend line represents the 95% confidence interval.

To validate the protocol, the RNA from different cultured viruses (DENV, ZIKV, YFV, WNV) was extracted and used in RT-PCR reactions with the Ampli-FlaCk primers and the OneStep RT-PCR Kit, which allowed both reverse transcription and PCR amplification to occur in the same reaction mix. Gel electrophoresis revealed clearly visible bands corresponding to the expected amplicons for each virus (Figure 2A and Figure S1). Then, the amplicons were barcoded and sequenced using a MinION (nanopore sequencing) device for 2 h. After basecalling, viral reads were analyzed using the Genome Detective Version 2.41 software [17], a user-friendly online tool that performs read mapping, consensus creation, and genotyping of viruses such as ZIKV, DENV, CHIKV, and YFV [16].

Sequencing results showed that the protocol can detect all eight viral species and generate nucleotide sequences for the successful discrimination of viral lineages (Table 1). This nanopore library yielded an average of 5682 reads for each sequenced virus, with a mean percentage of 1.75% of off-target reads (reads originating from random crosstalk between neighboring samples). An average of 58.84 reads/NTC ratio was also estimated for this experiment. This ratio indicates the sample’s number of reads on-target-normalized against the number of reads found in the no-template control, and a ratio > 10 was arbitrarily defined to indicate a reliable positive sample for the assigned virus, as previously suggested [19].

Serial dilutions of these eight viruses were also sequenced following the protocol conditions described earlier, and the results allowed us to estimate a preliminary limit of detection for the Ampli-FlaCk protocol, using the dilution’s Ct values as proxy indicators of viral load. The sequencing results showed that the highest Ct values, which generated enough data for reliable detection, ranged from 30 to 37 for dengue viruses, while CHIKV-diluted samples could be positively detected up to a Ct value of 40 (full results are listed in Table S3).

Known positive clinical specimens were selected to validate the Ampli-FlaCk protocol under the same reaction conditions described earlier. Extracted RNA from 14 DENV-1-, 13 DENV-2-, 14 CHIKV-, 1 ZIKV-, and 7 YFV-positive samples was subjected to multiplex RT-PCR treatment and sequenced on the MinION device for up to 6 h to maximize sequencing yield per sample. Analysis of the reads showed that the protocol was able to reliably identify the viral lineage in 83.67% (41/49) of the clinical samples (Table 2 and Table S4). Although some viral reads were detected in 8 samples (16.33%), the reads/NTC ratio for these samples was lower than the threshold to be consider a positive result. The median of 10,229 mapped reads per sample was calculated for the combined sequenced libraries, with long reads of ~1000 bases being recovered from 89.80% (44/49) of the samples. Plotting the number of mapped reads against Ct values, as a proxy indicator for viral load, showed a downward trend in the number of reads as Ct values increased (Figure 2B). As expected, similar trends were also observed in the depth-of-coverage and reads/NTC ratio plots (Figure 2C,D).

**Figure 3:**
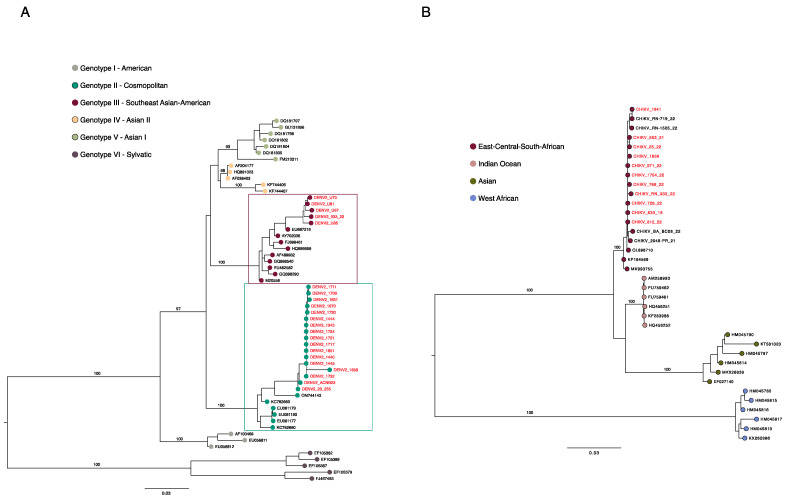
Reconstructed genotyping phylogeny of dengue virus type 2 (*Orthoflavivirus denguei)* and Chikungunya virus. (**A**) Maximum likelihood phylogenetic reconstruction of DENV-2 using partial sequences (n = 58) of the NS5 gene, including sequences (n = 22, red tips) generated in this study from clinical samples. (**B**) Maximum likelihood phylogenetic reconstruction of CHIKV using partial sequences (n = 36) of the envelope gene, including sequences (n = 11, pink tips) generated in this study from clinical samples. Numbers along branches represent Ultrafast bootstrap values (only for the main branches, to maintain clarity).

Once its efficacy to recover viral long reads from clinical samples had been established, the Ampli-FlaCk protocol was employed for testing in the field activities of an arbovirus genomic surveillance project carried out in Uruguay (early 2023) [20] and across Brazil in the years 2021 and 2022 [8]. The sequencing of 20 clinical samples collected during those surveillance activities resulted in the recovery and identification of reads of ZIKV, DENV-2, DENV-3, and DENV-4 (Table S5). It is worth mentioning that the protocol was also able to accurately recover reads of a recently detected lineage of DENV-2 in Brazil [21], the genotype II (cosmopolitan). To demonstrate that consensus sequences generated via the Ampli-FlaCk protocol can also be used to reconstruct phylogenetic trees accurately, we performed phylogenetic analyses using DENV-2 and CHIKV sequences from the positive clinical samples used in this study. The maximum likelihood tree showed that the new CHIKV sequences were grouped with other ECSA lineage isolates, while the new DENV-2 sequences were placed either in the genotype II–cosmopolitan clade or in the genotype III clade (Figure 3). Phylogenetic reconstructions were also performed for the other viruses sequenced in this study and can be seen in Supplementary Figures S2 and S3.

## 4. Discussion

DNA sequencing technologies are promising tools that can help in the laboratory detection of emerging and re-emerging pathogens, generating information that can contribute to the fight against epidemics [22,23]. In this study, we developed a protocol for the detection and genotyping of multiple viral species in a single reaction that combines the advantages of the high sensitivity and specificity of multiplex PCR with the portability and speed of data generation provided by nanopore sequencing, which can perform simultaneous sequencing of up to 96 samples in a single experiment. The Ampli-FlaCk protocol also incorporates the benefits of the OneStep RT-PCR Kit, which allows both reverse transcription and PCR amplification to take place in a single reaction [24].

Clinical samples were used for assessing the protocol performance, which resulted in viral identification of 83% of the samples. After sequencing, it was evident that the number of mapped reads followed a downward trend when Ct values increased. This negative relationship has been previously reported [25] for whole-genome sequencing of arboviruses from clinical samples and upholds the importance of sampling during the acute period of infections, when a high viral load is present. A Ct value of 37 is considered the limit of detection for dengue samples tested using the Trioplex Real-time RT-PCR Assay (CDC) [26]. Comparatively, the Ampli-FlaCk protocol was able to detect DENV sample dilutions with the highest Ct values ranging from 30 to 37, depending on the serotype, while CHIKV dilutions were detected up to a Ct value of 40. Although these results are encouraging and point to the potential of the protocol for detecting viral genetic material at low concentrations, the small number of samples tested limits our assessment of the effectiveness of the protocol when working with samples with a Ct value above 30. As such, additional testing is needed to determine the exact detection limit of the protocol.

The diagnosis of orthoflavivirus infections using serological methods is challenging due to the limitations imposed by the broad antigenic cross-reactivity among common orthoflaviviruses [27]. The protocol described here was shown to contribute to the successful detection of all orthoflaviviruses tested here by using long nucleotide sequences, which were also used for viral lineage assignments. Although this work did not test other viruses, the protocol has the potential to detect other orthoflavivirus species due to the use of primers with degenerate bases that cover the nucleotide diversity of the NS5 gene, as suggested by in silico analyses.

## 5. Conclusions

The protocol described in this study allowed us to obtain long reads of ~1000 bases from the NS5 and envelope genes of orthoflaviviruses and CHIKV, respectively. These reads can be used not only for the identification, but also for the classification of viral lineages. The obtained sequences can also be used for the reconstruction of phylogenetic trees and in evolutionary analysis that might help in understanding patterns of viral dispersion during an epidemic. The protocol practicality associated with the portability of the MinION device offers a possibility of using this approach in field studies in areas with limited infrastructure. Furthermore, the ability to wash and reuse the flow cells allows for a reduction in the costs of screening hundreds of samples when considering the protocol’s multiplex approach. Hence, the development of sequencing-based viral detection methods that are integrated into local epidemiological surveillance can be fostered in order to achieve a more anticipatory approach to epidemic prevention and control in public health [28].

## Figures and Tables

**Table 1:** Summary of sequencing statistics for the cultured viruses used for evaluating the performance of the Ampli-FlaCk protocol.

Sample	Ct	Number of Reads on Target	Off-Target Reads (%)	Reads/NTC Ratio	Lineage
DENV-1	25	4095	0.7	67.1	Genotype I
DENV-2	22	4058	0.8	66.5	Genotype III
DENV-3	19	4720	1.0	77.4	Genotype V
DENV-4	28	3390	2.3	55.6	Genotype II
YFV	28	4823	1.2	79.1	South America I
ZIKV	25	2449	1.4	40.1	Asian
WNV	33	3967	3.9	65.0	Lineage 1A
CHIKV	33	17954	2.8	19.8 *	ECSA
NTC	-	61	-	-	-
Mean (SD)		5682 (5015.23)	1.75 (1.14)	58.84 (20)	

SD = standard deviation; NTC = no-template control; * The CHIKV sample was sequenced using a separate library/flow cell; in this library, 905 reads were detected in the NTC.

**Table 2:** Summary of sequencing statistics for the clinical specimens used for evaluating the performance of the Ampli-FlaCk protocol.

Virus	# of Samples	Ct	# of Reads	% Off-Target Reads	Reads/NTC Ratio	Genotyping	Depth of Coverage ^1^	Sequence Length
DENV-1	14	19.49 (3.5)	31,754 (18,341.4)	0.01 (0.43)	590.41 (7896.47)	Genotype V	15,387.15 (12,991.5)	984.5 (38.3)
DENV-2	13	27.1 (3.6)	705 (9158.4)	4.33 (18.17)	80.10 (10,489.13)	Genotype III	2282.45 (4641.9)	1032.5 (109.5)
CHIKV	14	26.2 (4.6)	10,229 (18,977.3)	3.64 (31.15)	228.45 (6655.30)	ECSA	10,946.6 (21,163.4)	1051.5 (90.5)
YFV	7	16.8 (1.7)	41,143 (15,275.8)	0.14 (0.04)	1469.39 (545.57)	South America I	27,291 (9394.7)	1044 (49.5)
ZIKV	1	ND	62	0	62	Asian	30.4	960

Median values (standard deviation); ND = not detected; #=number. ^1^ Mean depth of coverage was estimated using SPAdes implemented via Genome Detective using the following function: read count * read length/contig length.

## Data Availability

The new sequences generated in this study have been deposited in NCBI GenBank under the accession numbers listed in Supplementary Table S6. Input data used for the phylogenetic analyses are provided in the repository https://doi.org/10.6084/m9.figshare.24270724.v2.

## Supplementary Materials

The following supporting information can be downloaded at https://www.mdpi.com/article/10.3390/v16010023/s1: Table S1. Nucleotide sequence and physical properties of the primers comprising the Ampli-FlaCk protocol. Table S2. List of potential orthoflaviviruses identified during in silico specificity assessment using MFEprimer. Figure S1. Validation of the Ampli-FlaCk primers on cultured viruses. Figure S2. Maximum Likelihood phylogenies reconstructed using the new sequences generated in this study and lineages reference sequences for genotyping. Figure S3. Maximum Likelihood phylogeny of yellow fever virus reconstructed using the new sequences generated in this study and lineages reference sequences for genotyping. Table S3. Sequencing results of serially diluted virus isolates. Table S4. Sequencing and genotyping results of clinical specimens used for validation of the Ampli-FlaCk protocol. Table S5. Sequencing and genotyping results of clinical specimens tested during viral genomic surveillance activities in Brazil and Uruguay. Table S6. List of GenBank access number of the sequences generated from the clinical samples in this study.
