# Supplementary material for: A Multiplex Nanopore Sequencing Approach for the Detection of Multiple Arboviral Species
Source: Viruses. 2023 Dec 22;16(1):23. doi: 10.3390/v16010023 (PMC10821003; doi:10.3390/v16010023)
Supplement: Supplementary file 1 [file viruses-16-00023-s001.zip › viruses-2753869-supplementary.pdf]

A multiplex nanopore sequencing approach for the detection of multiple arboviral species

Supplementary data

Table S1. Nucleotide sequence and physical properties of the primers comprising the Ampli-FlaCk protocol.

**Name**	Sequence	Length	Gene	Binding positions	GC content (%)	Melt temperature (mean)
Orthoflavi-rev	CATGTCDTCDGTNGTCATCCA	21	NS5	10054-10075	48.4	55.8 ºC
Flavi-all-S	TACAACATGATGGGGAARAGAGARAA	26	NS5	8993-9019	38.5	56.8 ºC
Flavi-all-S2	TACAACATGATGGGMAAACGYGARAA	26	NS5	8993-9018	40.4	59 ºC
CHIK_LEFT_1	CATGTACGCACCCATTTCACCA	22	E2	8913-8934	50	58.8 ºC
CHIK_RIGHT_0	CGGGATCACTGTTACGTGTTCG	22	E1	9997-10018	54.5	58.7 ºC

Primers physical properties were calculated using the OligoAnalyzer (Integrated DNA Technologies).
Binding positions refer to reference NC_002031 and KP164568.1 reference sequences for orthoflaviviruses (primes Orthoflavi-rev, Flavi-all-S, and Flavi-all-S2) and chikungunya virus (primers CHIK_LEFT_1 and CHIK_RIGHT_0), respectively.

Table S2. List of potential orthoflaviviruses identified during *in silico* specificity assessment using MFEprimer.

Virus name
dengue virus type 1
West Nile virus
dengue virus type 2
dengue virus type 3
Zika virus
Japanese encephalitis virus
Yellow fever virus
Tick-borne encephalitis virus
Louping ill virus
Alkhumra hemorrhagic fever virus
Bagaza virus
Powassan virus
Kyasanur Forest disease virus
Langat virus
Israel turkey meningoencephalomyelitis virus
Omsk hemorrhagic fever virus
Sepik virus
dengue virus type 4

Murray Valley encephalitis virus
Wesselsbron virus

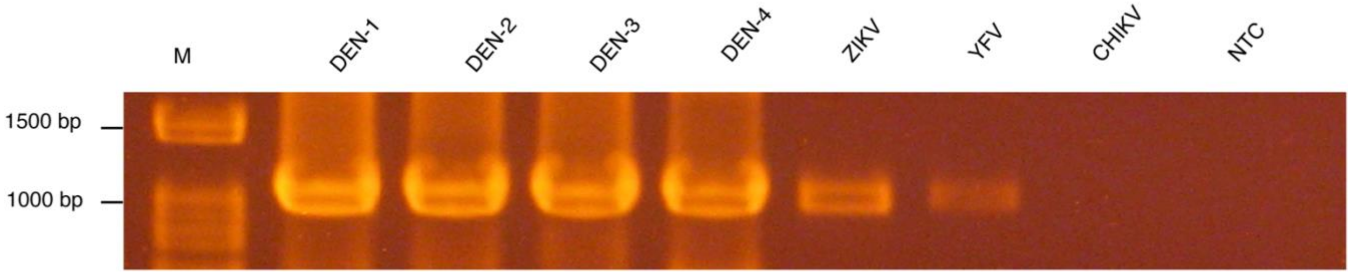

Figure S1. Validation of the Ampli-FlaCk primers on cultured viruses. RNA from cultured viruses (Chikungunya (CHIKV), dengue (DENV) 1 to 4, zika (ZIKV), yellow fever (YFV), and West Nile (WNV) viruses were used in RT-PCR with the three orthoflavivirus primers only (CHIKV primers were not included so specificity of the orthoflavivirus primers could be tested). Viruses presented the following RT-qPCR Ct values: DENV-1 (25), DENV-2 (22), DENV-3 (19), DENV-4 (28), CHIKV (33), YFV (28), and ZIKV (25). NTC= No Template Control.

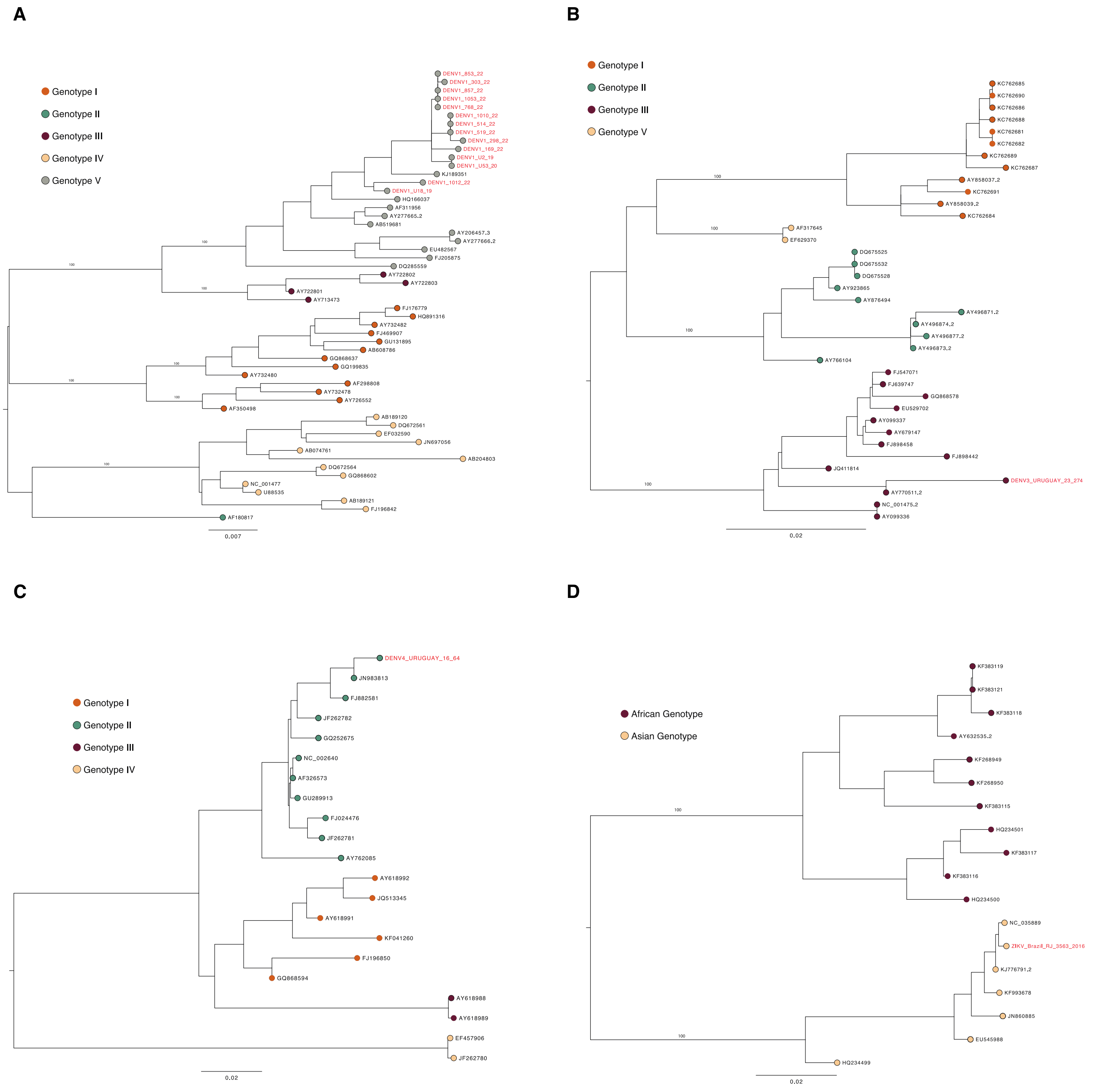

Figure S2. Maximum Likelihood phylogenies reconstructed using the new sequences generated in this study and lineages reference sequences for genotyping. A) Phylogeny of dengue virus serotype 1. New sequences' IDs (n=14) are colored in red. B) Phylogeny of dengue virus serotype 3. The new sequence's ID (n=1) is colored in red. C) Phylogeny of dengue virus serotype 4. New sequences' IDs (n=6) are colored in red. D) Phylogeny of zika virus. The new sequence's ID (n=1) is colored in red. A-D) Lineages are indicated by different colored circles at the tips of the branches.

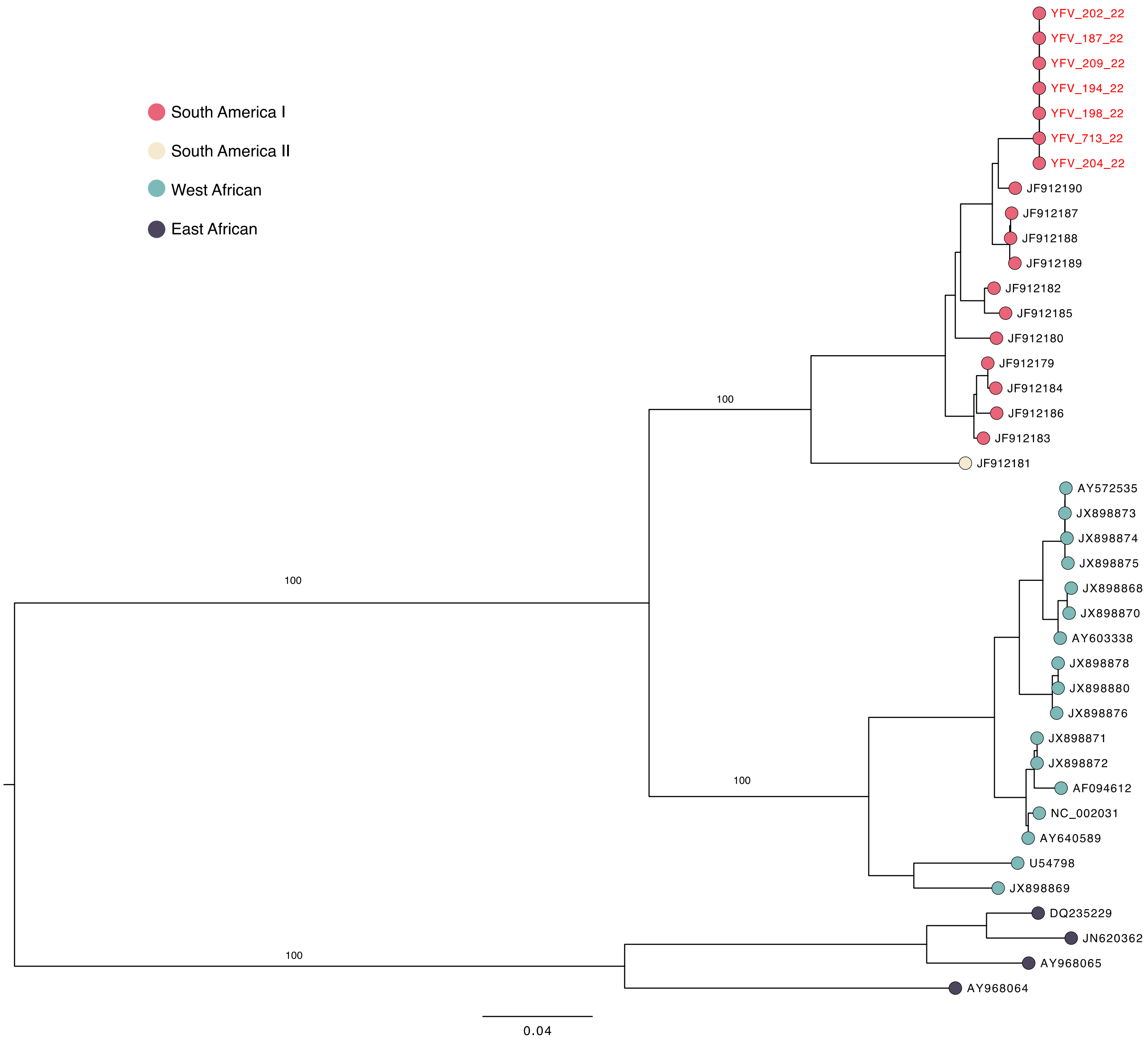

Figure S3. Maximum Likelihood phylogeny of yellow fever virus reconstructed using the new sequences generated in this study and lineages reference sequences for genotyping. New sequences' IDs (n=7) are colored in red. Lineages are indicated by different colored circles at the tips of the branches.

Table S3. Sequencing results of serially diluted virus isolates.

Virus	Ct	Dilution	Number of reads on target	reads/NTC ratio
DENV-1	27.1	10^{-3}	27651	141.8
	29.93	$2x10^{-4}$	13771	70.6
	37.07	***10^{-5}***	***546***	***91***
	40	10^{-6}	3	0.5
DENV-2	33.42	10^{-5}	6692	239
	33.39	***$2x10^{-6}$***	***1932***	***69***
	33.95	$13.3x10^{-7}$	13	0.5
DENV-3	30.98	10^{-4}	4198	149.9
	30.31	***$2x10^{-5}$***	***367***	***13.1***
	31.65	$13.33x10^{-6}$	57	2
DENV-4	30.05	$2x10^{-4}$	9954	51
	35.77	***10^{-5}***	***151***	***25.2***
	38.5	10^{-6}	1	0.2
ZIKV	***28.84***	***10^{-3}***	***3916***	***47.2***
	37.85	10^{-4}	250	3
	37.91	10^{-5}	512	6.2
CHIKV	35.9	10^{-1}	22110	172.7
	40.68	***10^{-2}***	***19525***	***152.5***
	ND	10^{-3}	78	0.6
YFV	***31.64***	***10^{-5}***	***9817***	***350.6***
	36.91	$2x10^{-7}$	18	0.6
	37.34	$4x10^{-8}$	19	0.7

ND=Not Detected
The detection limit for each virus is indicated by numbers highlighted in bold and was determined by a reads/NTC ratio > 10.

Table S4. Sequencing and genotyping results of clinical specimens used for validation of the Ampli-FlaCk protocol.

Library	Virus	Sample	Ct	Concentration [ng/µL][1]	Reads on target	off-target reads (%)	reads/NTC ratio	Genotyping	Bootstrap[2]	Depth[3]	Sequence length
1	DENV-2	854-18	28	0.53	5608	6.95	6.20	Genotype III	100	6162.4	971
	DENV-2	856-18	29	0.34	705	42.82	0.78	Genotype III	100	386.3	969
	DENV-2	1041-16	28	11.90	5081	6.56	5.61	Genotype III	100	3693.9	1044
	CHIKV	633-18	28	118.00	10504	4.72	11.61	ECSA	92.4	11715.7	1173
	CHIKV	553-21	37	5.42	9218	6.06	10.19	ECSA	88.2	7690.4	1056
	CHIKV	1092-18	ND	0.45	578	57.44	0.64	ECSA	88.2	366.8	1018
	NTC	NTC	-	0.00	905.0	-	-	-	-	-	-
2	DENV-1	U2	20	36.20	21792	1.59	21792.00	Genotype V	100	12706.5	993
	DENV-1	U18	16.8	18.90	12457	0.10	12457.00	Genotype V	100	8300.4	971
	DENV-1	U53	24.8	2.56	21072	0.56	21072.00	Genotype V	100	12630.2	976
	DENV-2	U61	NA	0.40	1120	2.10	1120.00	Genotype III	100	871	1021
	DENV-2	U67	23.4	5.56	18805	0.07	18805.00	Genotype III	100	11026.3	967
	DENV-2	U73	21.4	0.70	30119	0.07	30119.00	Genotype III	100	12228.6	1159
	DENV-2	U85	21.8	4.88	8621	0.47	8621.00	Genotype III	100	6057.8	1305
	NTC	NTC	-	0.20	0	-	-	-	-	-	-
3	CHIKV	1941	NA	3.14	75300	0.00	25100.00	ECSA	87.2	78885.7	1029
	CHIKV	1839	NA	7.20	9955	0.01	3318.33	ECSA	88	11739	1010
	CHIKV	**933**	29	13.60	13887	0.03	4629.00	ECSA	94.8	13017.8	1326
	DENV-2	**933**	25	13.60	462	0.86	154.00	Genotype III	100	57.4	1128
	NTC	NTC	-	0.352	3	-	-	-	-	-	-
4	DENV-1	169.22	18.3	86.20	32376	0.01	390.07	Genotype V	100	18142.3	994
	DENV-1	298.22	25.3	33.80	50287	0.01	605.87	Genotype V	98	2813.5	1072
	DENV-1	303.22	19	50.40	30634	0.03	369.08	Genotype V	100	18067.8	980
	DENV-1	514.22	18.7	63.20	19290	0.00	232.41	Genotype V	100	12325.3	986
	DENV-1	519.22	21.6	51.00	39341	0.00	473.99	Genotype V	100	2299.1	1053
	DENV-1	1010.22	21	63.20	47721	0.00	574.95	Genotype V	100	28465.2	969
	DENV-1	1012.22	15	87.80	34577	0.00	416.59	Genotype V	100	19409.8	978

Library	Virus	Sample	Ct	Concentration [ng/µL][1]	Reads on target	off-target reads (%)	reads/NTC ratio	Genotyping	Bootstrap[2]	Depth[3]	Sequence length
	DENV-1	1053.22	17	78.60	71161	0.00	857.36	Genotype V	100	43067.4	998
	DENV-1	853.22	21.7	95.40	31133	0.03	375.10	Genotype V	100	20268.2	980
	DENV-1	857.22	19	77.40	70155	0.00	845.24	Genotype V	99	41036.7	983
	DENV-2	745.22	29.4	0.00	0	-	-	-	-	-	-
	DENV-2	723.22	27.2	0.53	167	48.30	2.01	Genotype III	100	66.9	993
	DENV-2	1042.22	27	0.23	328	15.25	3.95	Genotype III	100	252.4	1115
	NTC	NTC	-	0.00	82	-	-	-	-	-	-
	DENV-2	1043.22	23.7	0.15	0	-	-	-	-	-	-
	DENV-2	1045.22	34	0.00	0	-	-	-	-	-	-
	CHIKV	25.22	22.7	90.60	1111	4.55	39.68	ECSA	85.1	1175	1042
	CHIKV	270.22	25.8	0.00	11	85.14	0.39	-	-	-	-
	CHIKV	571.22	22.5	82.60	1446	4.37	51.64	ECSA	86.1	1657.3	1047
	CHIKV	612.22	26.2	65.80	15233	2.93	544.04	ECSA	82	2325	1153
	CHIKV	726.22	23.4	108.00	11347	0.73	405.25	ECSA	83.4	15885.8	1085
	CHIKV	736.22	34	0.00	12	79.31	0.43	-	-	-	-
	CHIKV	1754.22	24.4	116.00	12778	0.67	456.36	ECSA	88.1	17090.3	1046
5	CHIKV	**768.22_C**	28.5	83.00	12103	0.22	432.25	ECSA	86.4	10177.5	1056
	DENV-1	**768.22_C**	27.3	83.00	18893	0.22	674.75	Genotype V	99	1677.7	1083
	YFV	187.22	16	78.80	41143	0.15	1469.39	South America I	100	27291	1096
	YFV	194.22	17.3	73.00	43711	0.08	1561.11	South America I	100	31319.8	1044
	YFV	198.22	15.7	85.00	33173	0.19	1184.75	South America I	100	22019.7	1041
	YFV	202.22	16.3	96.80	56720	0.11	2025.71	South America I	100	34273.5	1091
	YFV	204.22	20.8	39.00	66869	0.10	2388.18	South America I	100	44428.1	979
	YFV	209.22	17.6	104.00	26677	0.16	952.75	South America I	100	17354.4	1116
	YFV	713.22	16.8	72.80	26232	0.19	936.86	South America I	100	20264.3	1011
	NTC	NTC	-	0.00	28	-	-	-	-	-	-

Samples of codetection cases are highlighted in bold.
ND=Not Detected
NA= Not Available
NTC= No Template Control
[1]Sample concentration after PCR measured by Qubit.
[2]Sequence bootstrap support values obtained by virus genotyping tool from Genome Detective.
[3]Mean depth of coverage calculated by Genome Detective.

Table S5. Sequencing and genotyping results of clinical specimens tested during viral genomic surveillance activities in Brazil and Uruguay.

Location	Virus	Sample type	Sample ID	Collection date	Municipality	Ct	Number of reads on target	Mean depth of coverage	Nucleotide Identity (%)	Lineage
Acre - Brazil	DENV-2	Serum	ACNB23	2022-04-11	Xapuri	34.2	675	356.50	91.90	Genotype II - Cosmopolitan
Uruguay (Imported case from Mexico)	DENV-3	Serum	23-274	2023-04-29	Montevideo	25	6817	5493.80	93.10	Genotype III
Uruguay (Imported case from Paraguay)	DENV-4	Serum	16-64	2016-01-31	Montevideo	24	17916	6926.90	80.20	Genotype II
Uruguay (Imported case from Argentina)	DENV-2	Serum	23-255	2023-04-26	Montevideo	28	57800	12455.50	81.90	Genotype II - Cosmopolitan
Rio de Janeiro - Brazil	ZIKV	Serum	3563-16	2016	NA	IND	62	30.40	99.58	Asian
Amazonas - Brazil	DENV-2	Serum	1343	2022-08-08	Benjamin Constant	18	20991	728.08	92.37	Genotype II - Cosmopolitan
Amazonas - Brazil	DENV-2	Serum	1721	2022-09-10	Atalaia do Norte	21	14306	356.03	92.37	Genotype II - Cosmopolitan
Amazonas - Brazil	DENV-2	Serum	1440	2022-08-16	Benjamin Constant	27	5699	223.43	92.37	Genotype II - Cosmopolitan
Amazonas - Brazil	DENV-2	Serum	1970	2022-11-16	Atalaia do Norte	NA	13012	280.59	92.27	Genotype II - Cosmopolitan
Amazonas - Brazil	DENV-2	Serum	1711	NA	NA	NA	20968	471.42	92.97	Genotype II - Cosmopolitan
Amazonas - Brazil	DENV-2	Serum	1717	2022-07-10	Atalaia do Norte	22	14441	335.44	92.37	Genotype II - Cosmopolitan
Amazonas - Brazil	DENV-2	Serum	1724	2022-11-10	Atalaia do Norte	22	20661	522.95	92.62	Genotype II - Cosmopolitan
Amazonas - Brazil	DENV-2	Serum	1631	NA	NA	NA	6666	146.62	92.66	Genotype II - Cosmopolitan
Amazonas - Brazil	DENV-2	Serum	1851	2022-10-24	Benjamin Constant	NA	2755	56.70	92.37	Genotype II - Cosmopolitan
Amazonas - Brazil	DENV-2	Serum	1444	2022-08-26	Benjamin Constant	26	2174	85.82	92.37	Genotype II - Cosmopolitan
Amazonas - Brazil	DENV-2	Serum	1730	2022-11-10	Atalaia do Norte	24	20421	468.81	92.26	Genotype II - Cosmopolitan
Amazonas - Brazil	DENV-2	Serum	1709	NA	NA	NA	9248	204.79	92.47	Genotype II - Cosmopolitan

Location	Virus	Sample type	Sample ID	Collection date	Municipality	Ct	Number of reads on target	Mean depth of coverage	Nucleotide Identity (%)	Lineage
Amazonas - Brazil	DENV-2	Serum	1732	2022-10-10	Atalaia do Norte	24	10989	4059.90	92.50	Genotype II - Cosmopolitan
Amazonas - Brazil	DENV-2	Serum	1445	NA	NA	NA	3490	1256.40	92.20	Genotype II - Cosmopolitan
Amazonas - Brazil	DENV-2	Serum	1659	NA	NA	NA	48	13.30	82.30	Genotype II - Cosmopolitan

ND=Not Detected
NA= Not Available
NTC= No Template Control
Mean depth of coverage calculated by Genome Detective.

Table S6. List of GenBank access number of the sequences generated from clinical samples in this study.

**Sequence ID**	Access Number
CHIKV_633_18	OR578962
CHIKV_553_21	OR578960
CHIKV_1941	OR578968
CHIKV_1839	OR578967
CHIKV_RioGrandedoNorte_933_202	OR578969
CHIKV_25_22	OR578959
CHIKV_571_22	OR578963
CHIKV_612_22	OR578961
CHIKV_726_22	OR578964
CHIKV_1754_22	OR578966
CHIKV_768_22_C	OR578965
YFV_713_22	OR578977
YFV_209_22	OR578976
YFV_204_22	OR578974
YFV_202_22	OR578975
YFV_198_22	OR578973
YFV_194_22	OR578972
YFV_187_22	OR578970
ZIKV_RJ_3563_2016	OR506342

**Sequence ID**	Access Number
DENV1_U2	OR500978
DENV1_857_22	OR500979
DENV1_853_22	OR500980
DENV1_1053_22	OR500981
DENV1_1012_22	OR500982
DENV1_1010_22	OR500983
DENV1_519_22	OR500984
DENV1_514_22	OR500985
DENV1_303_22	OR500986
DENV1_298_22	OR500987
DENV1_169_22	OR500988
DENV1_768_22_C	OR500989
DENV1_U53	OR500990
DENV1_U18	OR500991
DENV2_ACRE_ACNB23_2022	OR500992
DENV2_Amazonas_1659	OR500993
DENV2_Amazonas_1445	OR500994
DENV2_URUGUAY_Montevideo_23_255	OR500995
DENV2_Amazonas_1732_2022	OR500996
DENV2_Amazonas_1343_2022	OR500997
DENV2_Amazonas_1721_2022	OR500998
DENV2_Amazonas_1440_2022	OR500999
DENV2_Amazonas_1970_2022	OR501000
DENV2_Amazonas_1711	OR501001
DENV2_Amazonas_1717_2022	OR501002
DENV2_Amazonas_1724_2022	OR501003
DENV2_Amazonas_1631	OR501004
DENV2_Amazonas_1851_2022	OR501005
DENV2_Amazonas_1444_2022	OR501006
DENV2_Amazonas_1730_2022	OR501007

**Sequence ID**	Access Number
DENV2_Amazonas_1709	OR501008
DENV2_U85	OR501009
DENV2_U73	OR501010
DENV2_U67	OR501011
DENV2_U61	OR501012
DENV2_933_22	OR501013
DENV3_URUGUAY_Montevideo_23_274	OR501014
DENV4_URUGUAY_Montevideo_16_64	OR501020
